# SiO_x_N_y_ back-contact barriers for CZTSe thin-film solar cells

**DOI:** 10.1371/journal.pone.0245390

**Published:** 2021-01-12

**Authors:** Wenjian Chen, Hippolyte Hirwa, Jörg Ohland, Teoman Taskesen, Ulf Mikolajczak, Devendra Pareek, Jürgen Parisi, Levent Gütay

**Affiliations:** Laboratory for Chalcogenide Photovoltaics, Energy and Semiconductor Research Laboratory, Institute of Physics, Carl von Ossietzky University of Oldenburg, Oldenburg, Lower Saxony, Germany; University of New South Wales, AUSTRALIA

## Abstract

The formation of molybdenum diselenide (MoSe_2_) is widely observed at the back-contact interface for copper zinc tin selenide (CZTSe) thin-film solar cells. Depending on individual selenium (Se) supply and thermal conditions for forming CZTSe absorbers on molybdenum (Mo) substrates, the thickness of MoSe_2_ can vary from a few hundreds of nanometers up to ≈ 1 μm, which is comparable to the commonly adopted thickness of 1 ~ 1.5 μm for CZTSe absorbers. In this study, for controlling the thickness of interfacial MoSe_2_, thin diffusion barrier layers of silicon oxynitride (SiO_x_N_y_) are deposited onto Mo layers prior to the growth of CZTSe absorbers in the fabrication process. As a result, a reduction in the thicknesses of MoSe_2_ layers is achieved. In terms of energy conversion efficiency (*η*), CZTSe solar cells grown on Mo/SiO_x_N_y_ back contacts suffer a deterioration as the SiO_x_N_y_ layers get thicker. CZTSe solar cells grown on Mo/SiO_x_N_y_/Mo back contacts preserve their efficiencies at ≈ 11% with thin 10 nm SiO_x_N_y_ layers.

## Introduction

Kesterite Cu_2_ZnSn(S,Se)_4_ (CZTSSe) is considered as a promising substitution for chalcopyrite Cu(In,Ga)(Se,S)_2_ (CIGSSe) in thin-film solar cell technology due to its earth abundant and low-cost constituents [1, 2]. However, in terms of the energy conversion efficiency (*η*), CZTSSe solar cells reach only 12.6% while CIGSSe devices have an up-to-date record of ≈ 23.4% [3, 4]. For pure Cu_2_ZnSnS_4_ and Cu_2_ZnSnSe_4_ solar cells, the record efficiencies are reported to be 11% and 12.5%, respectively [5, 6]. In order to further improve kesterite solar cells, addressing the back-contact issues is important, especially for pure CZTSe devices. By replacing CIGSSe with CZTSSe as the absorber material, molybdenum (Mo) is generally inherited as the back-contact material [7]. In most of the reported CZTSe synthesis processes, a MoSe_2_ layer with a thickness ranging from a few hundred nm up to ≈ 1 μm is observed at the Mo/CZTSe back-contact interface [8–10]. In general, the formation of such thick MoSe_2_ layers is considered to cause negative impacts on the device performance [10, 11]. And for the reported record 12.5% CZTSe solar cell, the MoSe_2_ thickness is at around 100 ~ 200 nm [6]. Therefore, the limiting and/or control of MoSe_2_ thickness at the back interface is commonly discussed as a possible way to improve the solar cell efficiency. Diffusion barriers are commonly adopted with back-contact structure Mo/barrier or Mo/barrier/Mo to avoid or suppress the formation of MoSe_2_ layers in kesterite solar cells [10, 12, 13]. As for silicon (Si) based microelectronic devices, silicon oxynitride (SiO_x_N_y_) is a widely used passivation material. With properties such as high temperature durability, high oxidation resistance and low defect density, it has the benefit of good availability in many research institutions [14, 15]. In this study, we deposit SiO_x_N_y_ layers as diffusion barriers with Mo/SiO_x_N_y_ and Mo/SiO_x_N_y_/Mo back-contact structures. For the as-grown CZTSe solar cells, we show and discuss the results in terms of back-interface morphology, solar cell performance and defect properties.

## Materials and methods

### Sample preparation

**Fig 1:**
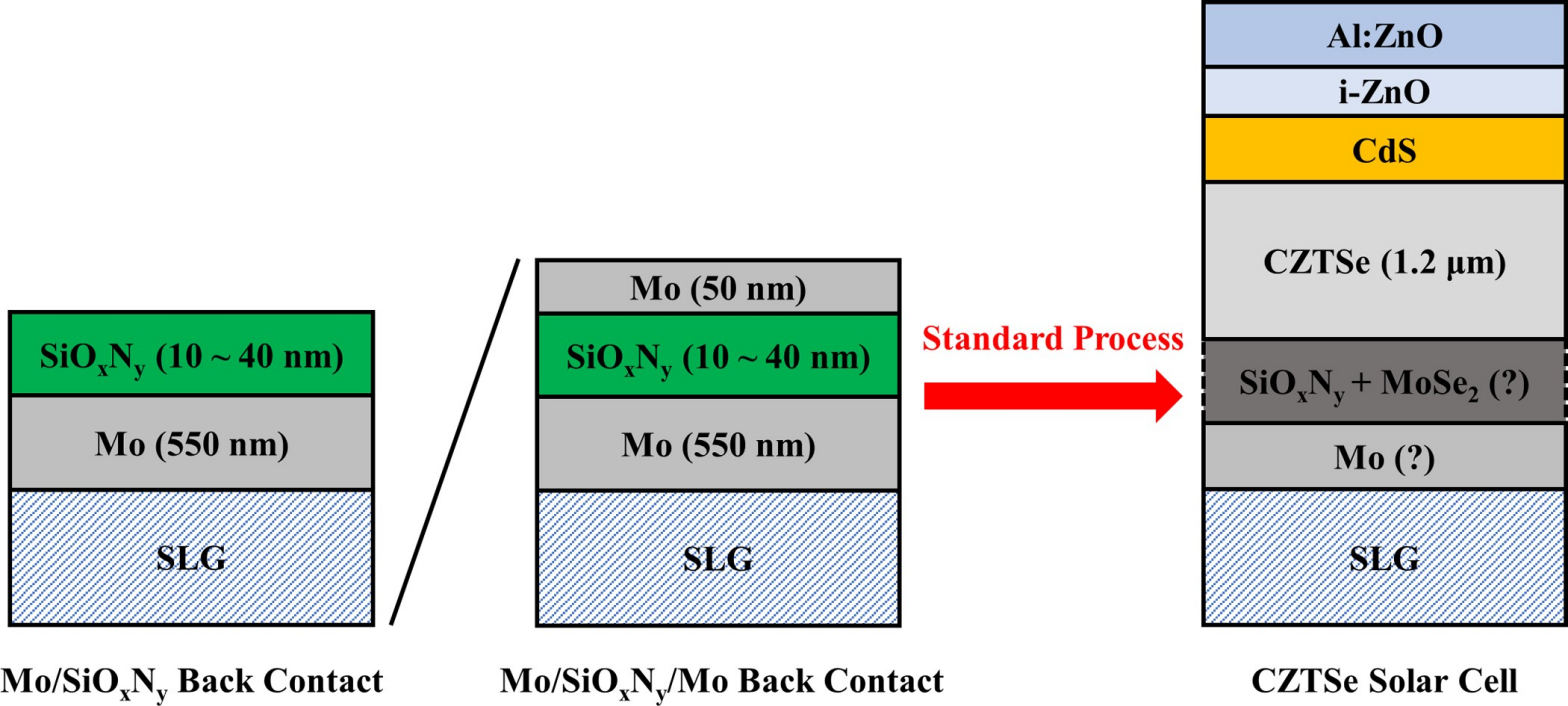
Schematic diagram of CZTSe solar cells grown with SiO_x_N_y_ back-contact barriers.

Two types of back-contact structures with SiO_x_N_y_ layers were applied: Mo/SiO_x_N_y_ and Mo/SiO_x_N_y_/Mo. As shown in Fig 1, a standard Mo layer (≈ 550 nm) consisting of two sub-layers (≈ 275 nm for each) was deposited by Ar plasma (power density: 6.1 W/cm^2^, pressure: 2.7×10^−3^ mbar) onto the 1 mm soda lime glass (SLG). SiO_x_N_y_ layers (10, 25 and 40 nm) were deposited from Si sputter target by mixed Ar-N_2_-O_2_ plasma (power: 160 W, pressure: 2×10^−3^ mbar) onto the standard Mo layers. For Mo/SiO_x_N_y_/Mo back-contact structure, the top Mo layer (≈ 50 nm) on SiO_x_N_y_ was deposited under the same conditions as for the standard Mo layers. For both back-contact structures, a standard procedure for the fabrication of solar cells in our lab was performed [8, 16, 17]. In this study, a dry cleaning with Ar plasma (power: 100 W, pressure: 5×10^−3^ mbar, duration: 90 s) was performed on the as-grown Mo/SiO_x_N_y_ and Mo/SiO_x_N_y_/Mo back contacts. For the formation of CZTSe absorber, a metallic precursor with a structure of Zn/Cu-Sn/Zn was deposited onto the above-mentioned back contacts by DC-sputtering at room temperature, followed by the annealing with selenium (Se) pellets and tin (Sn) wires in a tube furnace at 530°C for 20 minutes. As buffer layers, cadmium sulfide (CdS) was deposited onto the as-grown CZTSe absorbers (≈ 1.2 μm) via chemical bath. Furthermore, i-ZnO and Al:ZnO layers were deposited by RF-sputtering as front contacts. Finally, every sample was divided by mechanical scribing into solar cells with an average area of ≈ 0.25 cm^2^. In the following statement, the reference solar cell with a standard 550 nm Mo back contact is denoted by ‘‘M”. The solar cells grown on Mo/SiO_x_N_y_ back contacts are denoted by ‘‘MS10”, ‘‘MS25” and ‘‘MS40”, for which the thicknesses of SiO_x_N_y_ layers are 10, 25 and 40 nm, respectively. ‘‘MM” denotes the reference solar cell with a Mo/Mo back contact, in which a top layer of Mo (≈ 50 nm) is deposited on the standard Mo layer. ‘‘MS10M”, ‘‘MS25M” and ‘‘MS40M” denote solar cells grown on Mo/SiO_x_N_y_/Mo back contacts, in which the thicknesses of SiO_x_N_y_ layers between the top and the standard Mo layers are 10, 25 and 40 nm, respectively.

### Characterization

A FEI Helios Nanolab 600i scanning electron microscope (SEM) was used for the characterization of cross-section morphology. A Keithley 2400 SMU was adopted for current-voltage (*I*-*V*) measurements of CZTSe solar cells under standard AM 1.5 illumination in a PET SS100AAA solar simulator. A Bentham PVE300 system was used for EQE measurements. Measurements of capacitance-frequency (*C*-*f*) and thermal admittance spectroscopy (TAS) were performed with a Solartron impedance analyzer SI-1260. The admittance spectra were recorded for a frequency range from 10 Hz to 1 MHz and a temperature range from 50 K to 330 K. The processes of heating and cooling were performed in a closed cycle Helium cryostat at a base pressure < 10^−5^ mbar. For precise measurements of the temperature, a thermal sensor was glued on top of a dummy cell placed next to the real sample.

## Results and discussion

### Mo/SiO_x_N_y_ back contact

**Fig 2:**
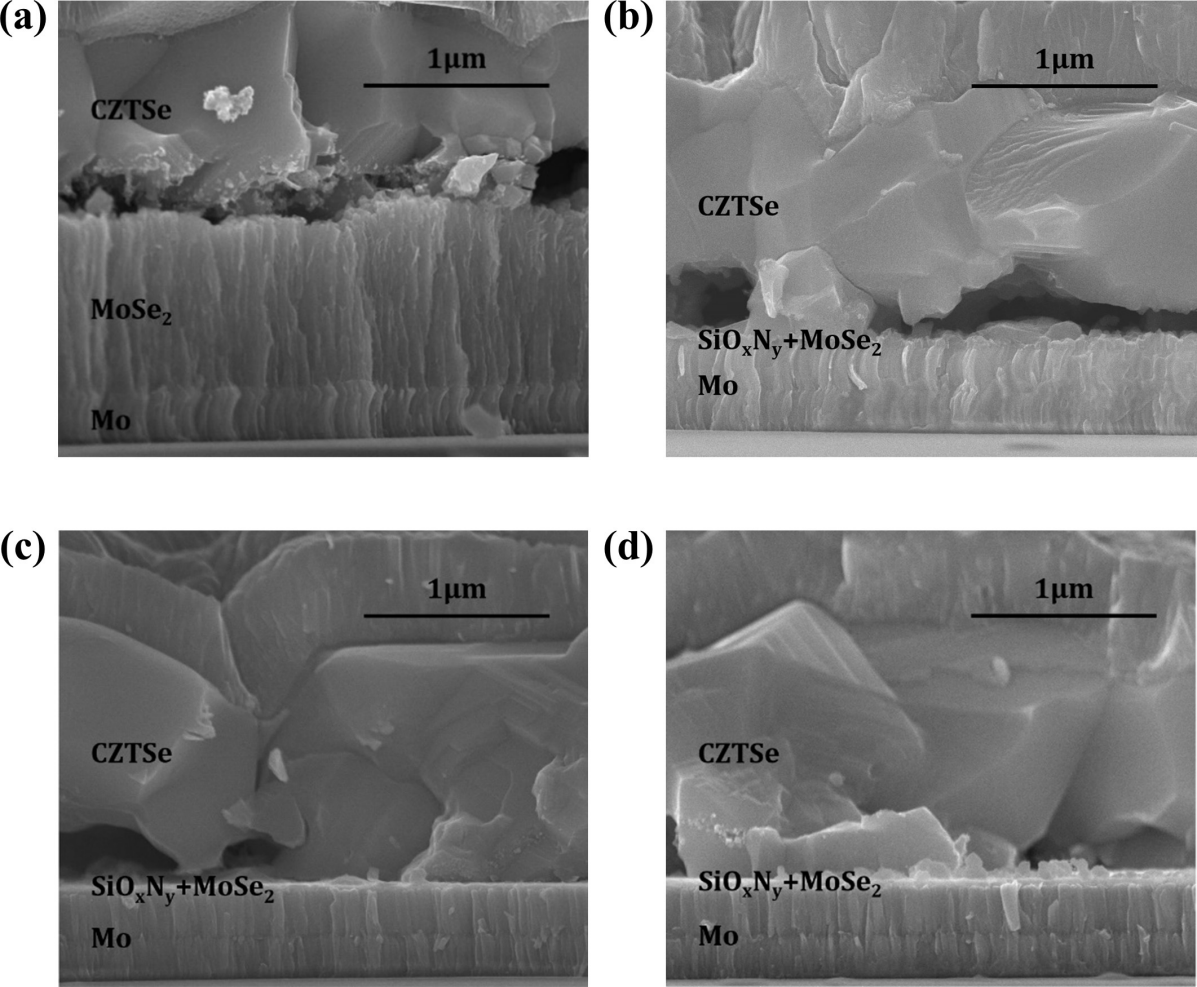
SEM cross-section of CZTSe solar cells grown on Mo and Mo/SiO_x_N_y_ back contacts. Images are captured for samples **(a)** M, **(b)** MS10, **(c)** MS25 and **(d)** MS40, respectively.

Fig 2 shows SEM cross-section morphology of CZTSe samples grown on Mo and Mo/SiO_x_N_y_ back contacts. The reference sample M, which has no SiO_x_N_y_ diffusion-barrier layer, shows the formation of a ≈ 1 μm MoSe_2_ interfacial layer. Samples with SiO_x_N_y_ layers in various thicknesses (i.e., 10, 25 and 40 nm) show a significantly suppressed formation of MoSe_2_ layers. The specific thicknesses of formed MoSe_2_ layers lie in the range of 30 ~ 40 nm with no visible trend. This suggests that SiO_x_N_y_ acts as an effective diffusion barrier for Se and prevents the strong reaction of Mo and Se to form MoSe_2_ during a high temperature (≈ 530°C) annealing.

**Fig 3:**
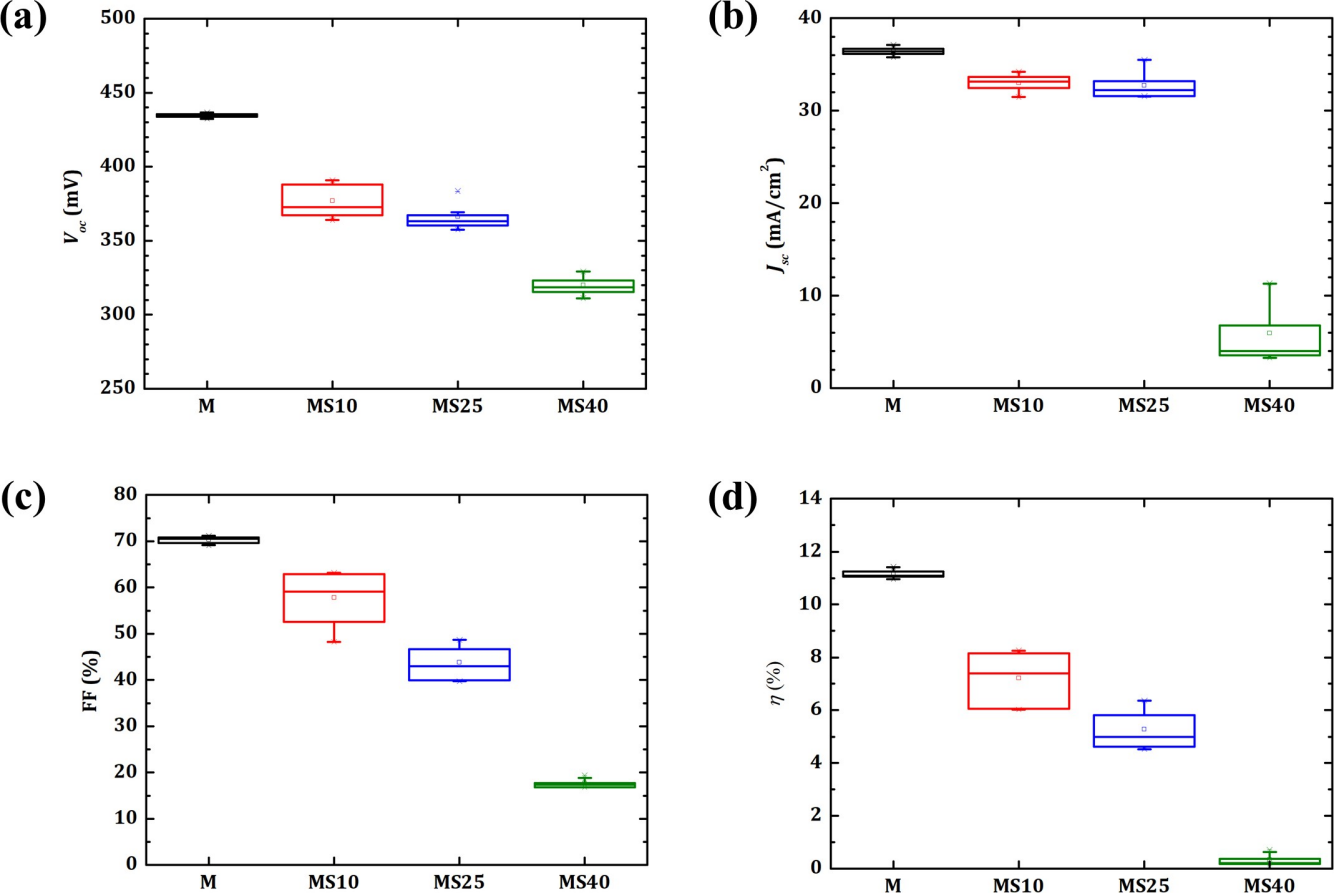
Parameters of CZTSe solar cells grown on Mo and Mo/SiO_x_N_y_ back contacts. Boxplots of solar cell parameters **(a)** *V*_*oc*_, **(b)** *J*_*sc*_, **(c)** FF and **(d)** *ŋ* for every type of back contacts include data from 6 to 9 cells.

Performance of the CZTSe solar cells grown on Mo and Mo/SiO_x_N_y_ back contacts are shown in Fig 3. Compared to the reference M, which is grown on pure Mo back contact, all the solar cells from Mo/SiO_x_N_y_ back contacts show a deterioration in all parameters, i.e., open-circuit voltage (*V*_*oc*_), short-circuit current density (*J*_*sc*_), fill factor (FF) and energy conversion efficiency (*η*). The reasons for the observed deterioration of solar cell properties may include a possible formation of an extra potential barrier induced by SiO_x_N_y_ layers. Furthermore, it cannot be excluded that a small amount of oxygen atoms from SiO_x_N_y_ may diffuse into the CZTSe absorbers and lead to additional impurity states in the absorber, which could impact the defect landscape or influence the phase structure of kesterite material in relevant regions. Moreover, thicker SiO_x_N_y_ diffusion barriers may further cause a harmful influence on the device performance by blocking sodium diffusion from SLG, which is generally considered to enhance the absorber quality in CIGS and kesterite solar cells [18–20]. However, based on our previous research, in which SiO_x_N_y_ was investigated as barrier layers between SLG and Mo, a total blocking effect for sodium was only achieved with much thicker SiO_x_N_y_ layers [21]. That means the blocking effect of SiO_x_N_y_ layers for sodium have most probably only a very minor influence in the present case. As a consequence, the CZTSe solar cells grown on Mo/SiO_x_N_y_ back contacts show overall poor performance, regardless of a possible positive effect expected from the reduced thicknesses of MoSe_2_ layers.

**Fig 4:**
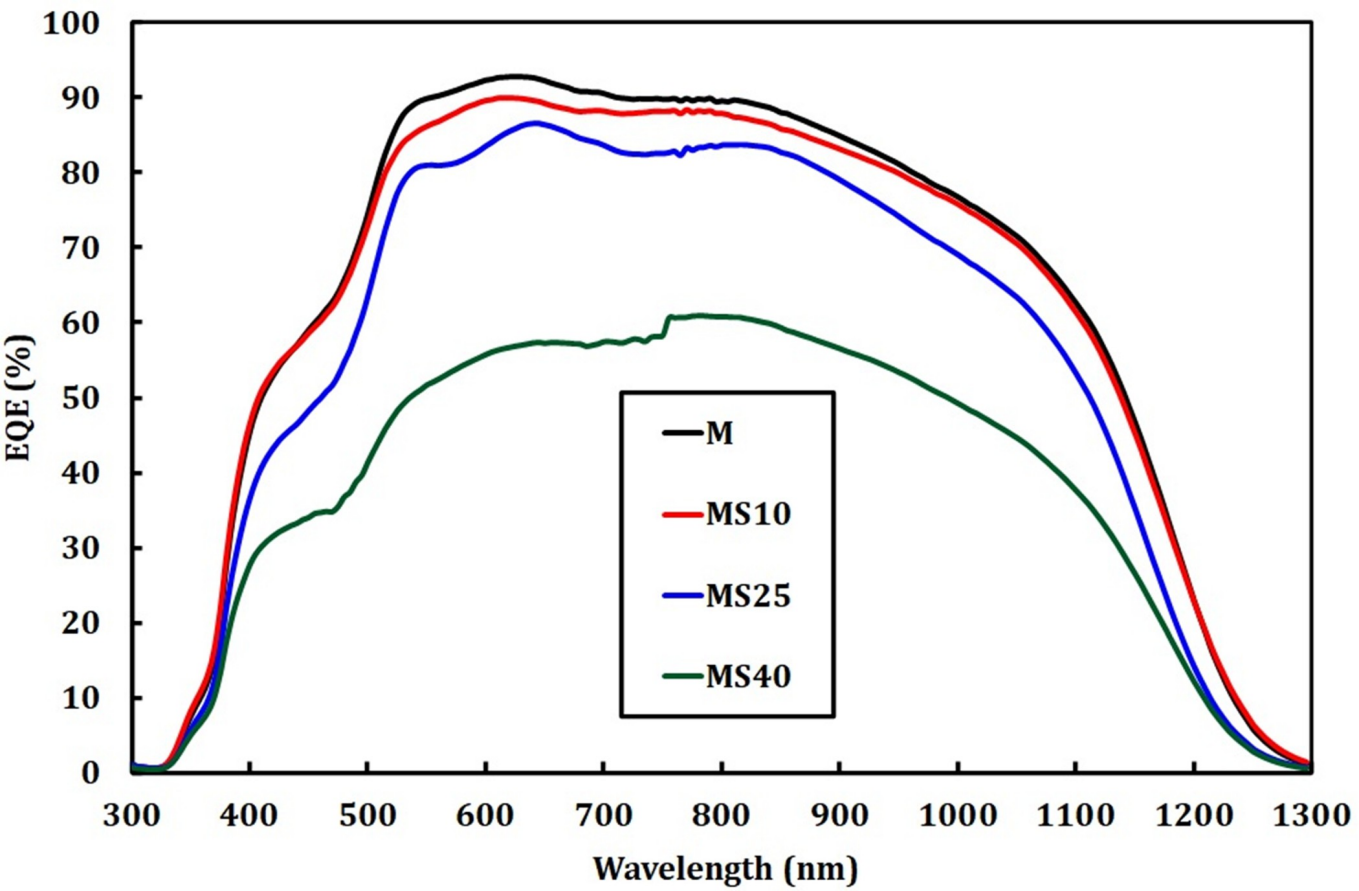
EQE measurements of CZTSe solar cells grown on Mo and Mo/SiO_x_N_y_ back contacts.

Fig 4 shows the EQE of CZTSe solar cells grown on Mo and Mo/SiO_x_N_y_ back contacts. The overall EQE drops for the samples with SiO_x_N_y_ layers compared to that of the reference. Specifically, MS10 and MS25 show a slight drop while MS40 shows a strong one. This result matches the performance shown in the previous *I*-*V* measurements. In particular for the sample MS40, the strong drop in EQE and in the extracted short-circuit current (S1 Fig) suggest not only an increased series resistance, for which the EQE drop is wavelength independent, but also a potential barrier introduced by the thick SiO_x_N_y_ layer and/or a deterioration in absorber quality.

**Fig 5:**
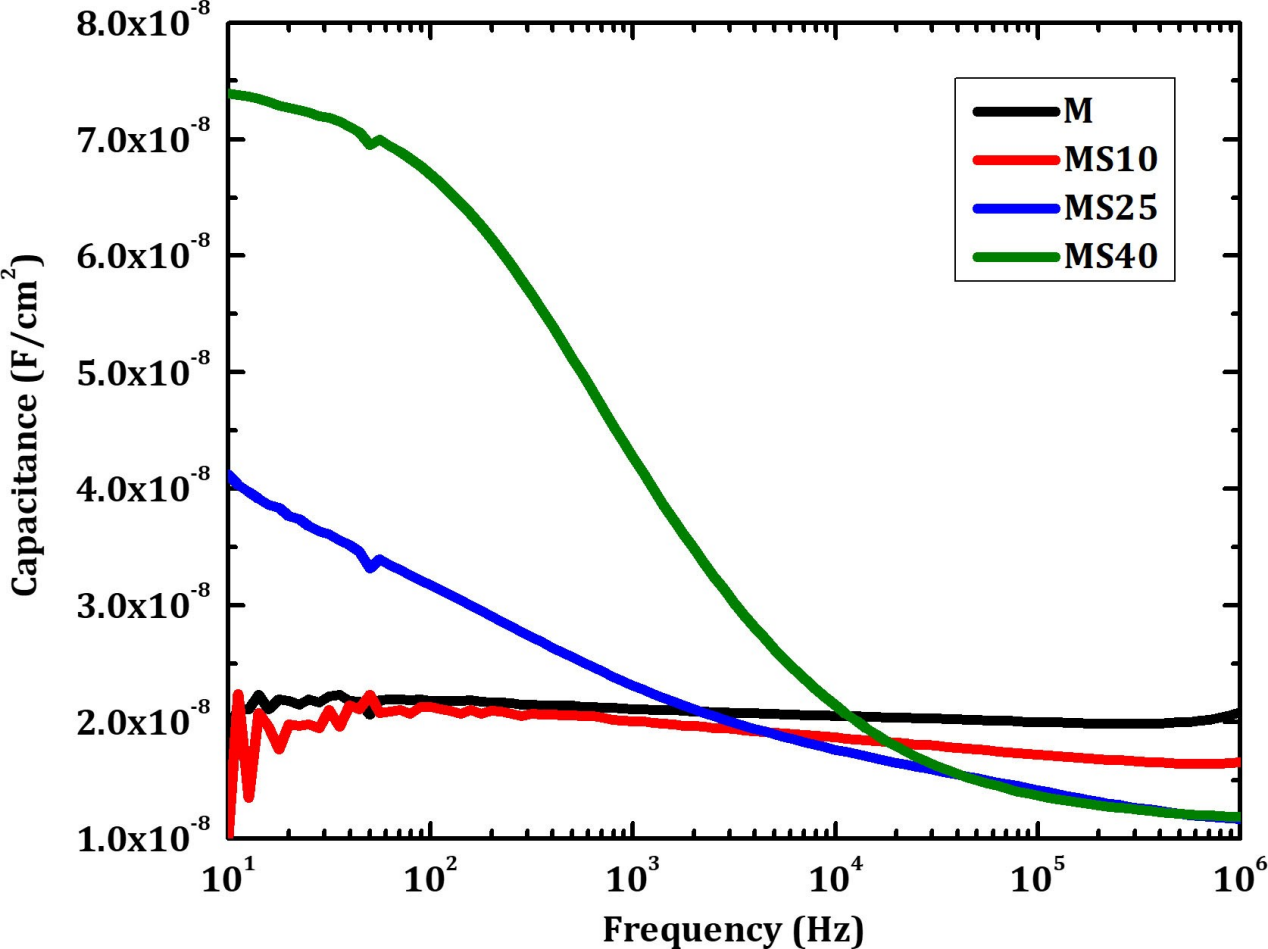
*C-f* measurements of CZTSe solar cells grown on Mo and Mo/SiO_x_N_y_ back contacts.

Fig 5 shows results from *C-f* measurements of CZTSe solar cells grown on Mo and Mo/SiO_x_N_y_ back contacts. According to literature about similar material-systems, inflection points related to shallow defects can be observed in capacitance measurements between 10 Hz and 100 kHz at temperatures between 50 K and 200 K [22–25]. In our case, samples M and MS10 do not show clear inflection points. Samples MS25 and MS40, which are grown on back contacts with thicker SiO_x_N_y_ layers, show clear inflection points. This may suggest a higher defect-density for CZTSe solar cells grown on Mo/SiO_x_N_y_ back contacts with the thicker SiO_x_N_y_ layers. Specific distributions of defects are investigated by TAS measurements and shown in the following part.

**Fig 6:**
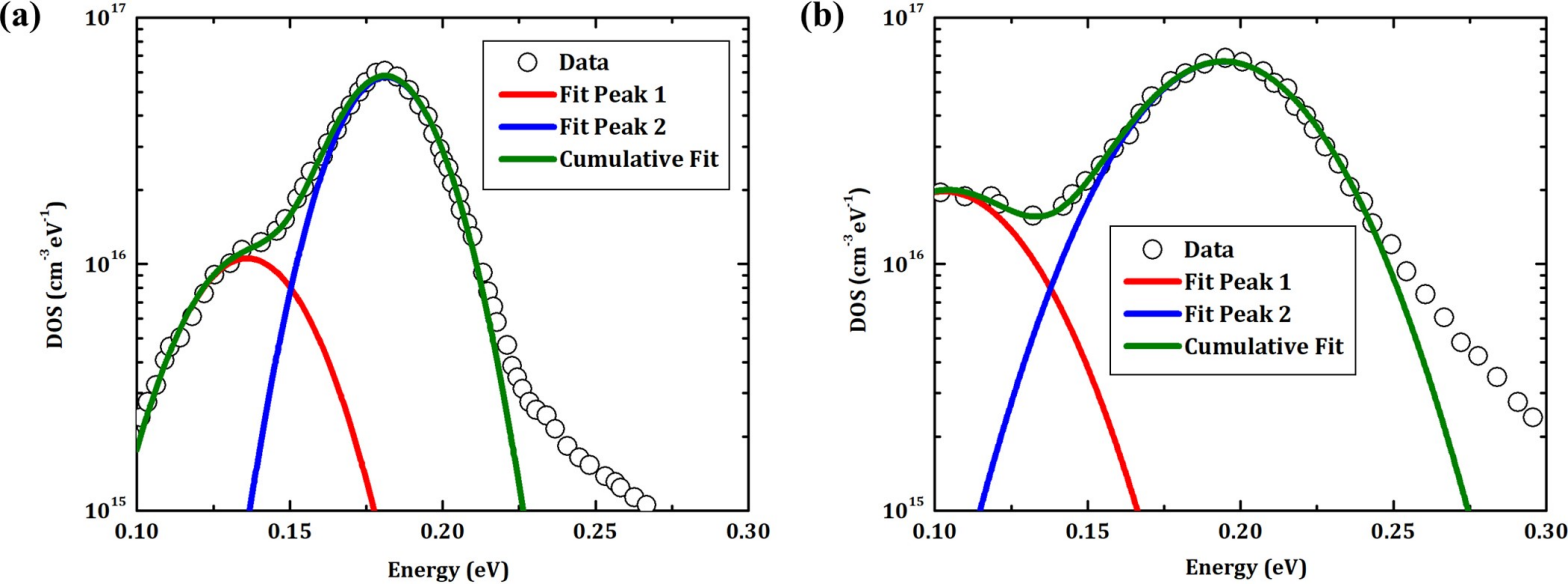
Density of states for CZTSe solar cells grown on Mo and Mo/SiO_x_N_y_ back contacts. DOS are derived from TAS measurements on samples **(a)** M and **(b)** MS40, respectively.

Density of states (DOS) derived from TAS measurements on CZTSe solar cells M and MS40 are shown in Fig 6. The results of sample M, as shown in Fig 6A, reveal a small peak at ≈ 0.13 eV and a large peak at ≈ 0.18 eV. For sample MS40, as shown in Fig 6B, both peaks are broadened in comparison to the previous case and their maxima are shifted to ≈ 0.10 eV and ≈ 0.20 eV, respectively. According to literature based on Cu_2_ZnSn(S,Se)_4_, both peaks may be linked to bulk defects [22, 25]. The significant broadening of the deeper defect is visibly accompanied by an enhanced density of states at deeper levels, which could cause higher recombination rates and thus a deterioration of solar cell properties.

### Mo/SiO_x_N_y_/Mo back contact

**Fig 7:**
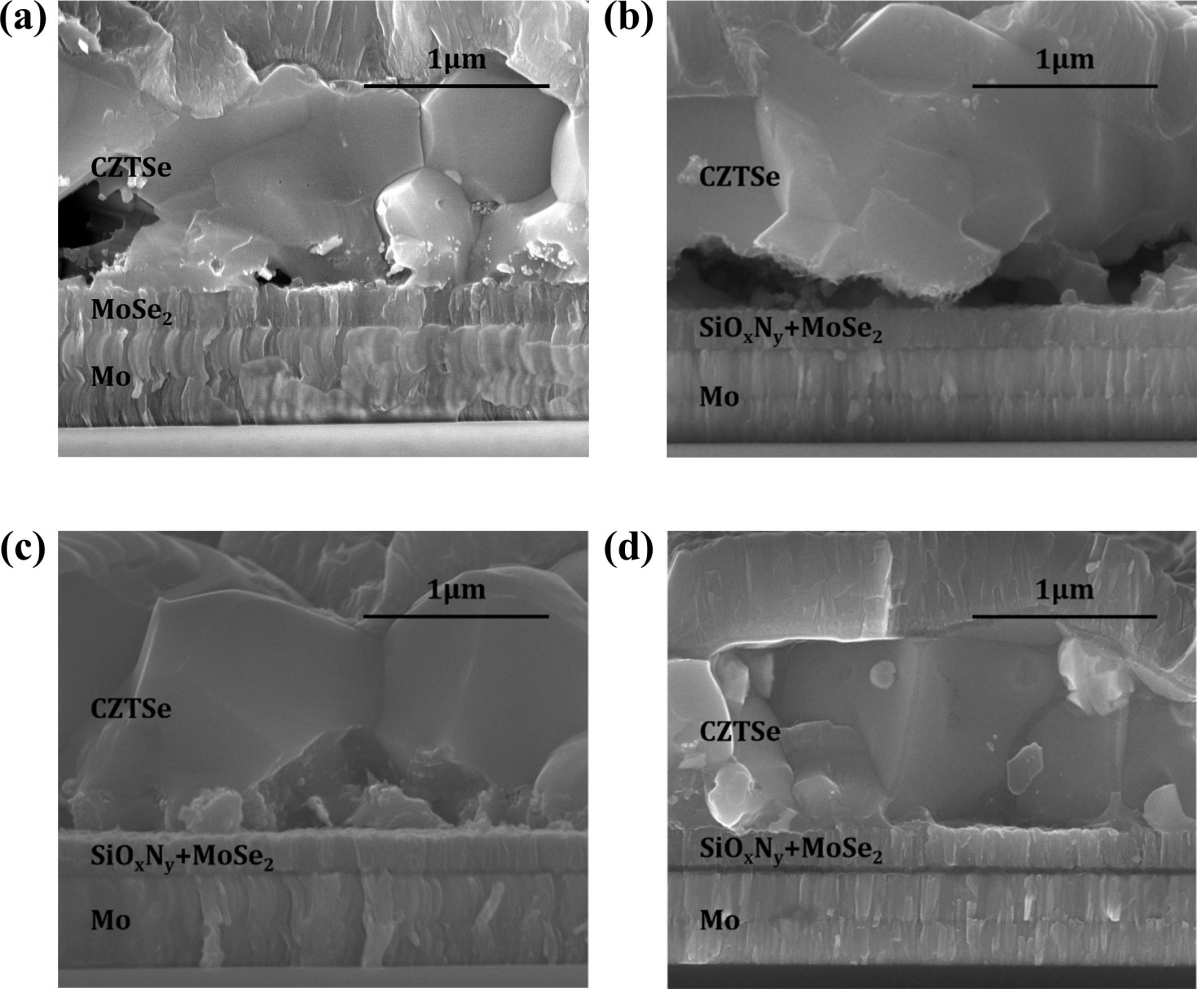
SEM cross-section of CZTSe solar cells grown on Mo/Mo and Mo/SiO_x_N_y_/Mo back contacts. Images are captured for samples **(a)** MM, **(b)** MS10M, **(c)** MS25M and **(d)** MS40M, respectively.

Cross-section morphology of CZTSe solar cells grown on Mo/Mo and Mo/SiO_x_N_y_/Mo back contacts is shown Fig 7. In comparison to the case of sample M, the thicknesses of interfacial MoSe_2_ layers of MS10M, MS25M and MS40M are reduced to a range of 230 ~ 240 nm with no obvious trend for the thicknesses of the investigated SiO_x_N_y_ layers. It indicates that only the top Mo layers (≈ 50 nm) in Mo/SiO_x_N_y_/Mo structures contribute to the formation of MoSe_2_ layers during the annealing and the Mo layers (≈ 550 nm) underneath SiO_x_N_y_ barriers remain intact. Surprisingly, for the reference sample MM, in which no barrier is applied, the thickness of interfacial MoSe_2_ stays also in a similar range as for the samples grown on Mo/SiO_x_N_y_/Mo back contacts. This indicates a barrier-like behavior at the Mo/Mo interface, which could be related to a natural passivation due to the process-break in Mo fabrication or a blocking effect due to crystal discontinuity in this layered structure.

**Fig 8:**
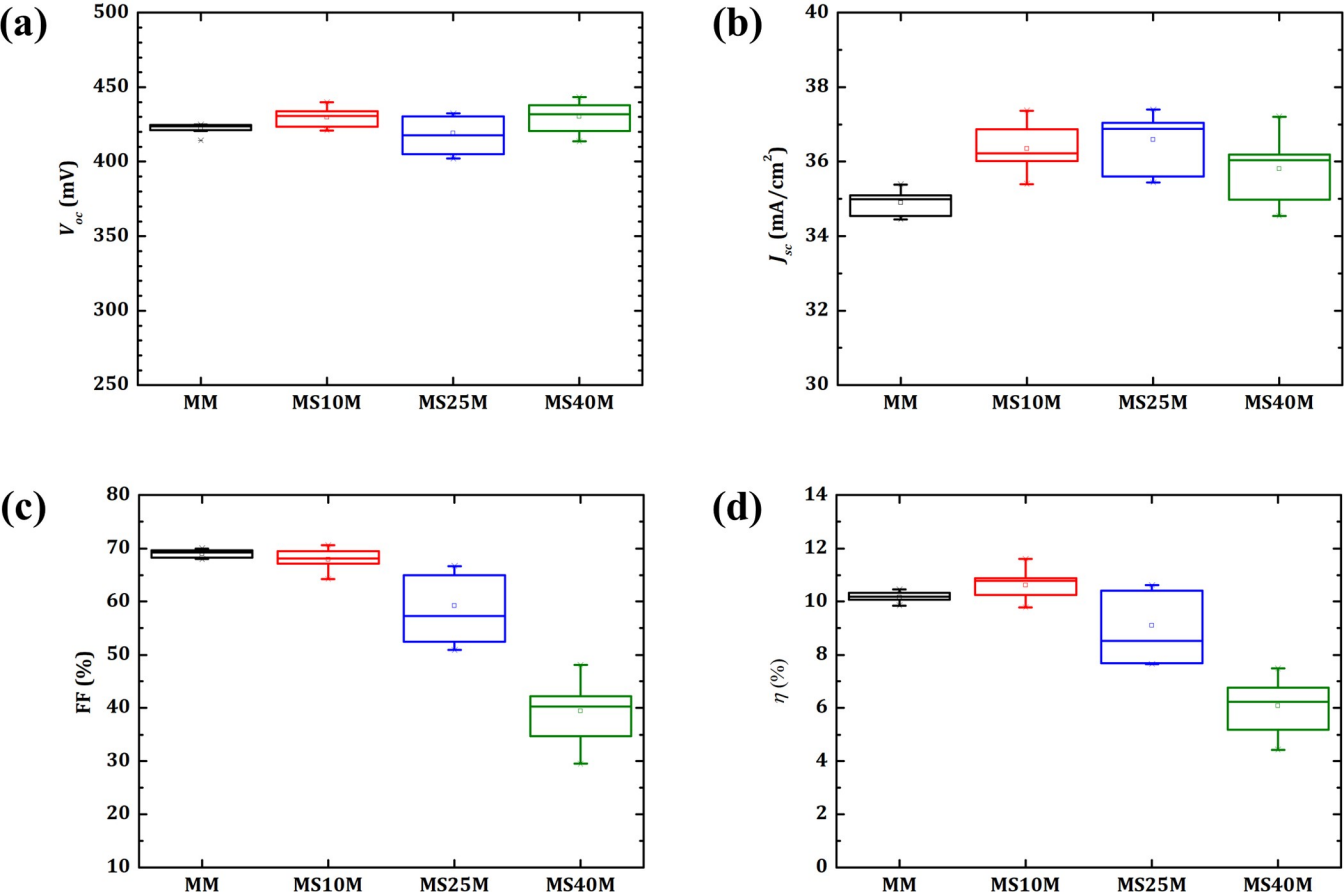
Parameters of CZTSe solar cells grown on Mo/Mo and Mo/SiO_x_N_y_/Mo back contacts. Boxplots of solar cell parameters **(a)** *V*_*oc*_, **(b)** *J*_*sc*_, **(c)** FF and **(d)** *ŋ* for every type of back contacts include data from 6 to 9 cells.

Fig 8 shows parameters of CZTSe solar cells grown on Mo/Mo and Mo/SiO_x_N_y_/Mo back contacts. For the sample MS10M, all the device parameters (*V*_*oc*_, *J*_*sc*_, FF and *ŋ*) stay in a similar or slightly improved range in comparison to the reference MM. In cases of thicker SiO_x_N_y_ layers (MS25M and MS40M), the device performance deteriorates. However, the deterioration here may differ from the samples grown on Mo/SiO_x_N_y_ back contacts. In cases of Mo/SiO_x_N_y_ back contacts, the deterioration could possibly be a combined result from the negative influence of SiO_x_N_y_ at the back interface and the deteriorated CZTSe absorber quality. For Mo/SiO_x_N_y_/Mo back contacts, a thin SiO_x_N_y_ layer may have a positive effect due to its role as passivation layer in between the formed porous MoSe_2_ and the intact bottom Mo, which could compensate the disadvantages such as causing an electrical barrier or an extra series resistance. As a result, for MS10M, without a noticeable drawback in FF, a similar *V*_*oc*_ and a small increase in *J*_*sc*_ lead to an improved *ŋ* at around 11% in comparison to the case of MM. And compared to M, MS10M gives a similar *ŋ* with a higher homogeneity in the morphology at the back interface. However, for the cases of MS25M and MS40M, in which the SiO_x_N_y_ layers are thicker, the possible negative effects of SiO_x_N_y_ on the back interface and the CZTSe absorber quality dominate and cause a significant drop in FF thus the *ŋ* of the whole devices.

**Fig 9:**
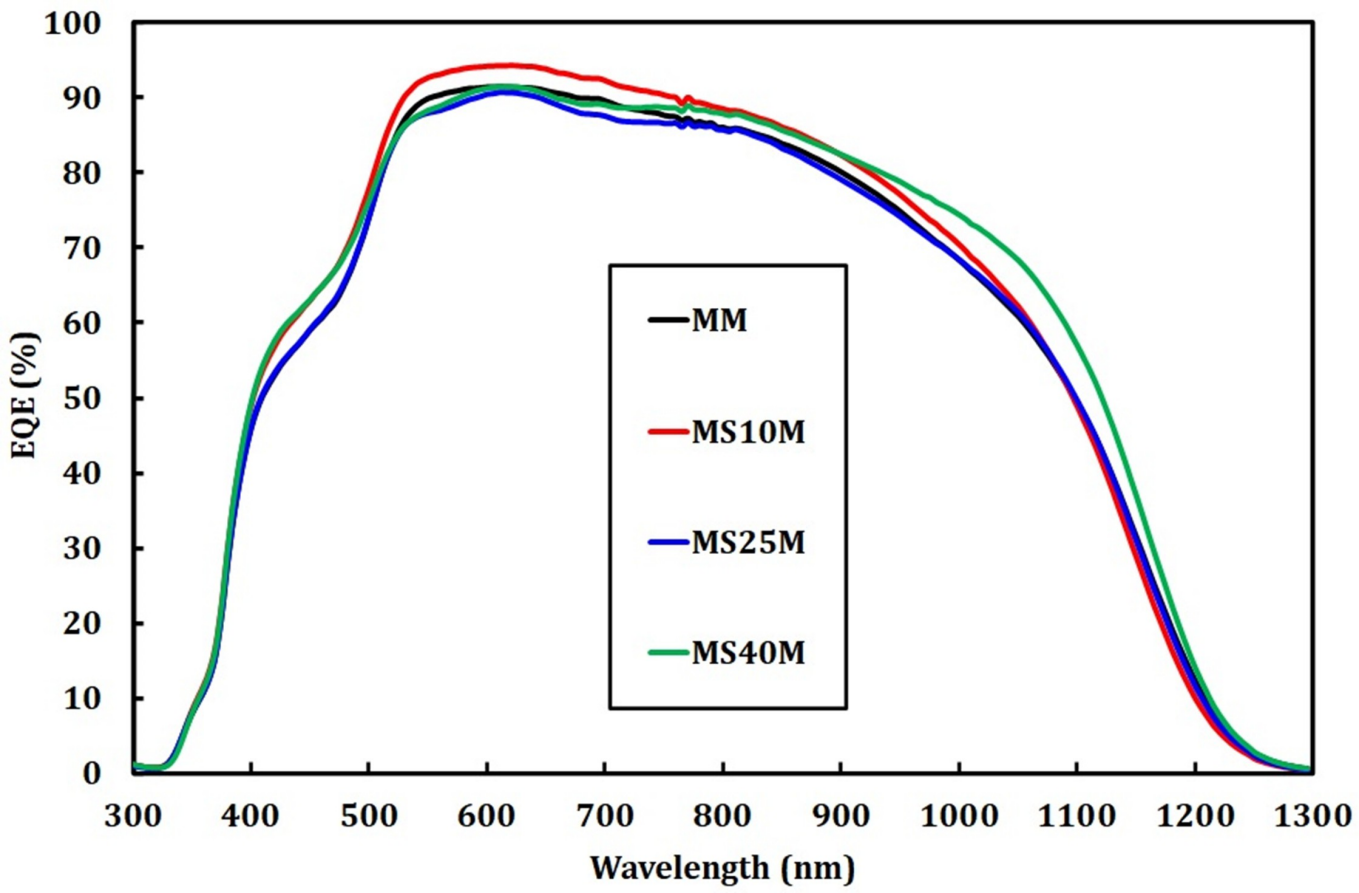
EQE measurements of CZTSe solar cells grown on Mo/Mo and Mo/SiO_x_N_y_/Mo back contacts.

Fig 9 shows EQE of CZTSe solar cells grown on Mo/Mo and Mo/SiO_x_N_y_/Mo back contacts. Compared to that of the previous samples grown on Mo/SiO_x_N_y_ back contacts, the change in thicknesses of the added SiO_x_N_y_ layers has a much smaller impact on the EQE, which shows no clear trend. This indicates that the negative effects of a thick SiO_x_N_y_ layer are significantly reduced by applying a 50 nm Mo layer on top. EQE of all devices reach ≈ 90% at around 600 nm. The extracted values of *J*_*sc*_ remain constant at around 35 mA/cm^2^ (S2 Fig).

**Fig 10:**
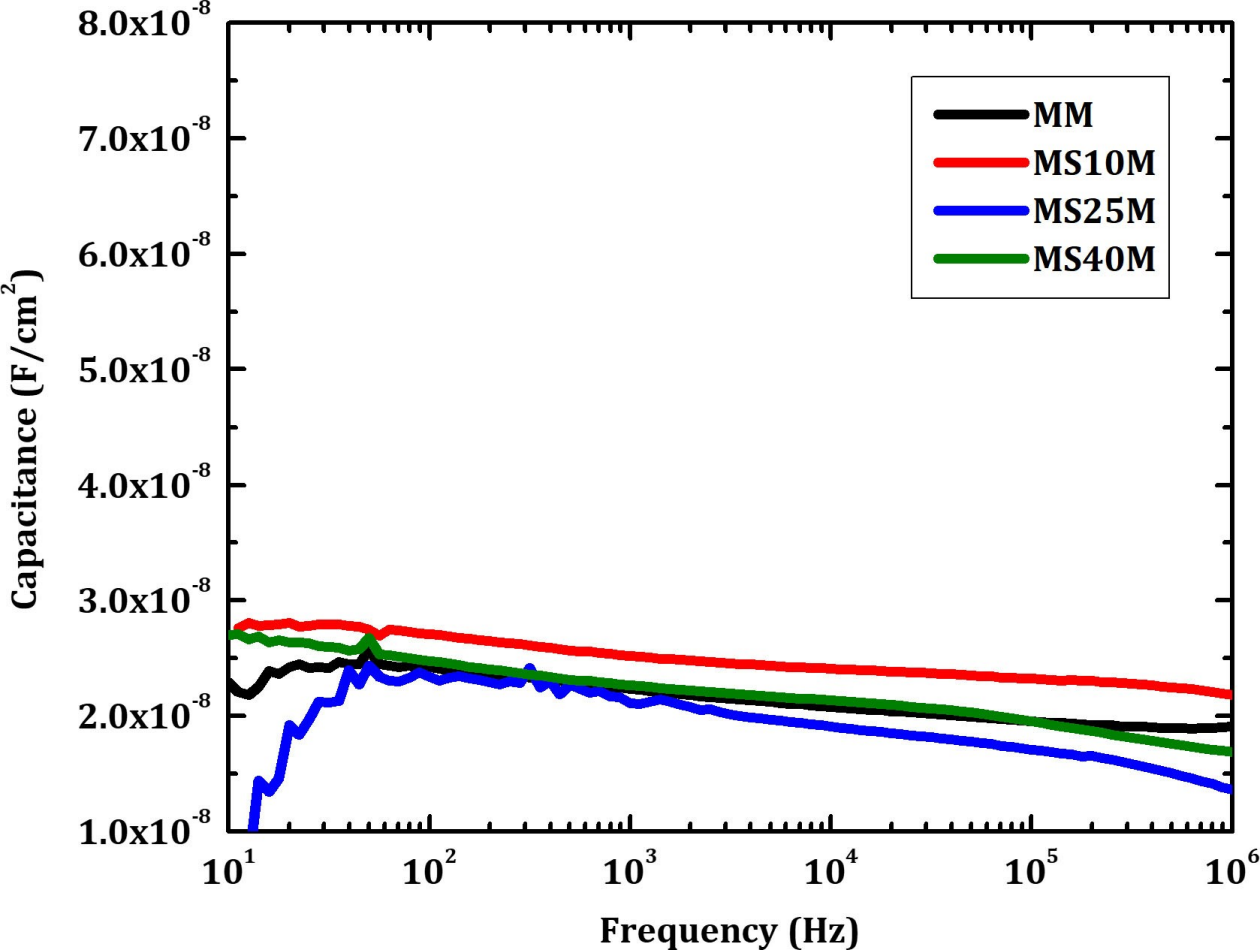
*C-f* measurements of CZTSe solar cells grown on Mo/Mo and Mo/SiO_x_N_y_/Mo back contacts.

Fig 10 shows *C-f* measurements of CZTSe solar cells grown on Mo/Mo and Mo/SiO_x_N_y_/Mo back contacts. Compared to that of the samples without the 50 nm Mo top layers, no clear inflection point is observed for all the samples grown on Mo/SiO_x_N_y_/Mo back contacts. This result is consistent with the above-discussed *I-V* and EQE measurements, suggesting that the additional Mo layers on top of the SiO_x_N_y_ can suppress or compensate the negative impacts from SiO_x_N_y_ layers. The presence of MoSe_2_ may have beneficial effects on the overall interface quality due to a more favorable alignment of the work functions and hence the bands of the adjacent layers.

**Fig 11:**
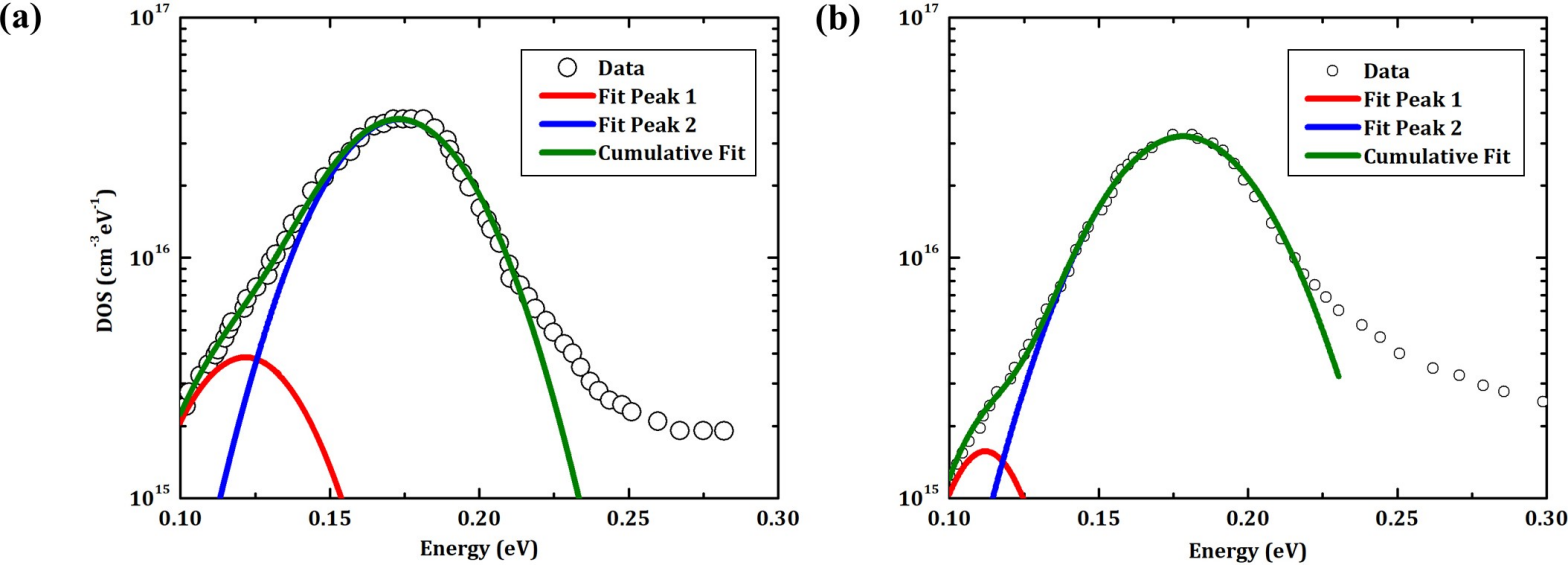
Density of states for CZTSe solar cells grown on Mo/Mo and Mo/SiO_x_N_y_/Mo back contacts. DOS are derived from TAS measurements on samples **(a)** MM and **(b)** MS40M, respectively.

Fig 11 shows the DOS derived from TAS measurements of the CZTSe solar cells MM and MS40M. In comparison with Fig 6, the overall influence of SiO_x_N_y_ on DOS is much smaller. In details, compared to the case of MM, the small peak at ≈ 0.12 eV is reduced and the large peak at ≈ 0.18 eV is slightly broader for MS40M. If the shallow levels at ≈ 0.12 eV is related to the interface defects, this result could indicate that SiO_x_N_y_ is beneficial for suppressing interface defects. However, the slight broadening of the deep levels at ≈ 0.18 eV still may indicate a negative influence of SiO_x_N_y_ on raising CZTSe bulk defects. As a result, the combined outcome from the changes at back interface and in absorber bulk is reflected in the device performance.

## Conclusions

Our study shows that SiO_x_N_y_ can act as an effective diffusion barrier for Se, thus significantly suppressing the formation of MoSe_2_ at Mo/CZTSe back-contact interface. For CZTSe solar cells grown on Mo/SiO_x_N_y_ back contacts, device parameters deteriorate with the increasing thicknesses of SiO_x_N_y_ layers. The incorporation of a SiO_x_N_y_ barrier layer could not only influence the Mo/CZTSe back-contact interface but also the CZTSe absorber. In cases of Mo/SiO_x_N_y_/Mo back contacts, the performance of CZTSe solar cells remain unchanged or slightly improved in the range of ≈ 11% for the adoption of 10 nm SiO_x_N_y_ layers. As the SiO_x_N_y_ layers gets thicker, the efficiencies of the solar cells decrease much less in comparison to the Mo/SiO_x_N_y_ cases. Overall, rather than the MoSe_2_ thickness, the behavior of back-contact interfaces as well as the absorber quality seem to be the crucial factors influencing the performance of kesterite solar cells.

## Supporting information

S1 Fig *J*_*sc*_ extracted from EQE measurements of CZTSe solar cells grown on Mo and Mo/SiO_x_N_y_ back contacts. (TIF)

S2 Fig *J*_*sc*_ extracted from EQE measurements of CZTSe solar cells grown on Mo/Mo and Mo/SiO_x_N_y_/Mo back contacts. (TIF)
