# Supplementary figures and images for: SiOxNy back-contact barriers for CZTSe thin-film solar cells
Source: PLoS One. 2021 Jan 12;16(1):e0245390. doi: 10.1371/journal.pone.0245390 (PMC7802926; doi:10.1371/journal.pone.0245390)

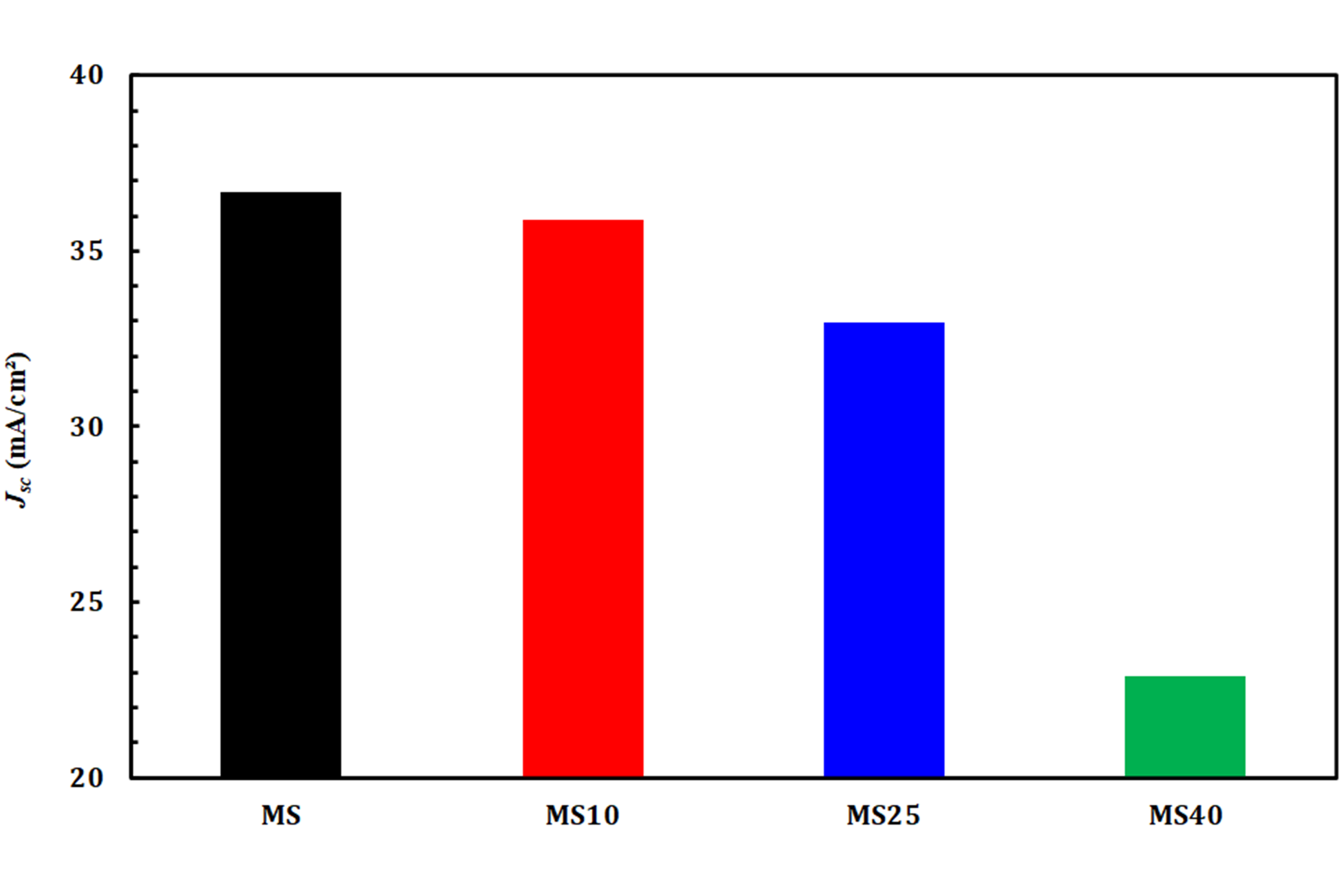

Supplement: S1 Fig — (TIF) [file pone.0245390.s001.tif]

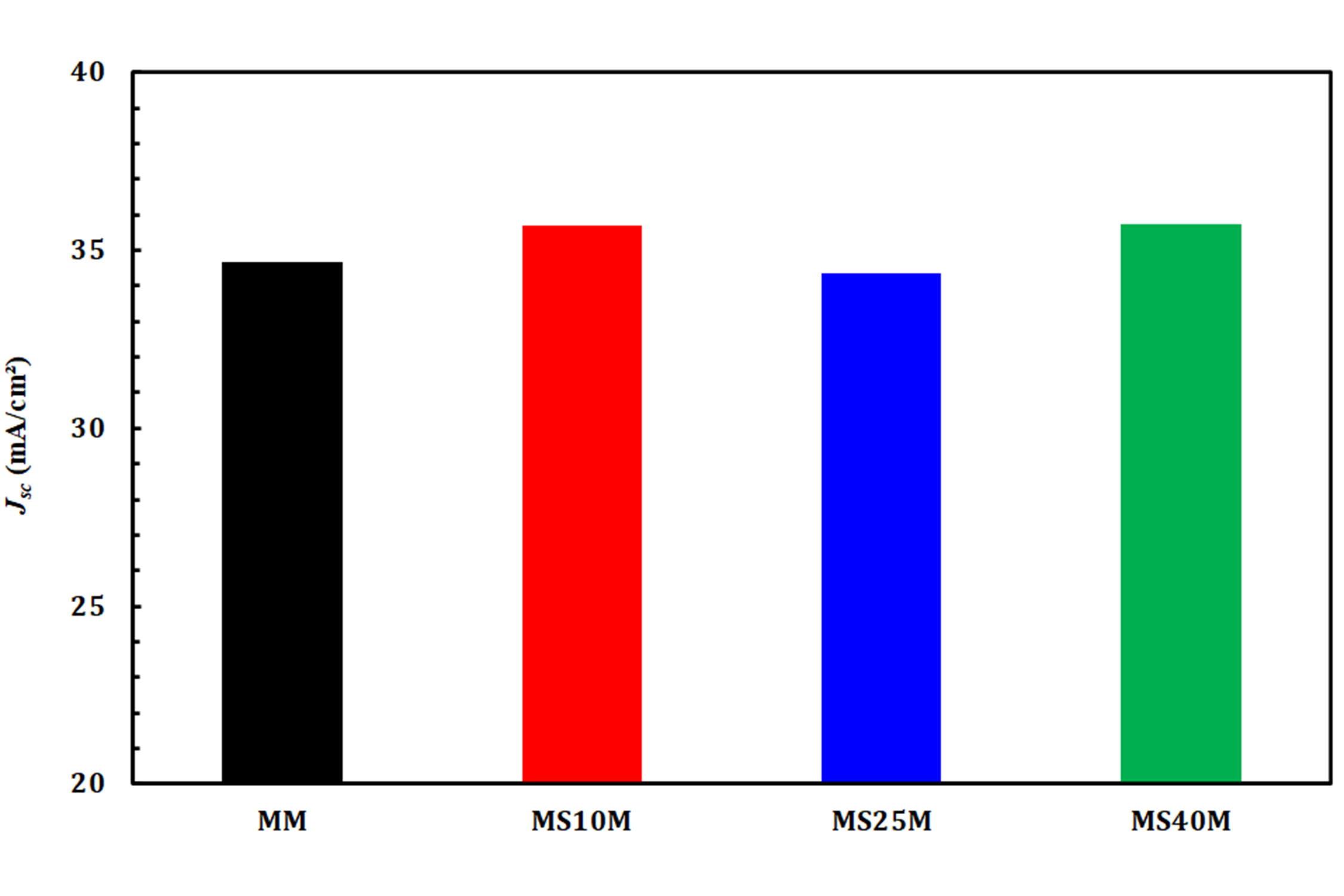

Supplement: S2 Fig — (TIF) [file pone.0245390.s002.tif]
